# Effectiveness of a web-based self-help smoking cessation intervention: protocol of a randomised controlled trial

**DOI:** 10.1186/1471-2458-9-32

**Published:** 2009-01-22

**Authors:** Jeannet JAM Kramer, Marc C Willemsen, Barbara Conijn, Andrée J van Emst, Suzanne Brunsting, Heleen Riper

**Affiliations:** 1 Innovation Centre of Mental Health and Technology (ICOM), Trimbos Institute, PO Box 725, 3500 AS Utrecht, the Netherlands; 2 STIVORO, PO Box 16070, 2500 BB Den Haag, the Netherlands

## Abstract

**Background:**

Cigarette smoking is a major risk factor for many chronic and fatal illnesses. Stopping smoking directly reduces those risks. The aim of this study is to investigate the effectiveness of a web-based interactive self-help programme for smoking cessation, known as the StopSite, by comparing it to an online self-help guide. Both interventions were based on cognitive-behavioural and self-control principles, but the former provided exercises, feedback and interactive features such as one-to-one chatrooms and a user forum, which facilitated mutual support and experience sharing.

**Methods and design:**

We conducted a randomised controlled trial to compare the interactive intervention with the self-help guide. The primary outcome measure was prolonged abstinence from smoking. Secondary outcomes were point-prevalence abstinence, number of cigarettes smoked, and incidence of quit attempts reported at follow-up assessments. Follow-up assessments took place three and six months after a one-month grace period for starting the intervention after baseline. Analyses were based on intention-to-treat principles using a conservative imputation method for missing data, whereby non-responders were classified as smokers.

**Discussion:**

The trial should add to the body of knowledge on the effectiveness of web-based self-help smoking cessation interventions. Effective web-based programmes can potentially help large numbers of smokers to quit, thus having a major public health impact.

**Trial registration:**

ISRCTN74423766

## Background

Smoking heightens the risks for many diseases, such as lung cancer [1], throat cancer [2], obstructive pulmonary disease (COPD) [3,4]and cardiovascular diseases [5]. It strongly increases mortality as expressed in lost life-years, and it is the most prominent risk factor for such mortality [6,7]. On average, smokers die about 10 years younger than non-smokers [7]. The World Health Organization (WHO) attributed 5.4 million deaths in 2005 to tobacco use, a figure expected to rise to 6.4 million a year by 2015 [8]. Stopping smoking is known to reduce the hazards of former smokers and increase their life expectancy. The earlier they quit, the more life-years they gain [7].

In view of the pernicious consequences for smokers and those around them and the positive effects of quitting, smoking cessation is an important topic on the political agenda. A current Dutch government policy aim is to reduce the percentage of smokers in the population from 28% in 2007 to 20% in 2010 [9]. Yet smokers are known to have a low likelihood of quitting: more than half of all current smokers want to stop, but less than 7% of quit attempts result in long-term abstinence. This may be partly due to the insufficient utilisation of effective help and support programmes [10].

STIVORO is a Dutch non-profit agency whose primary aim is to help people stop smoking. It offers a wide range of effective smoking cessation methods in a stepped-care approach. Smokers are advised to begin with less intensive, less costly methods (self-help guide, tailored advice) that do not involve intervention by a professional. If these do not work, more intensive forms of support are provided (coaching by telephone, group training, individual counselling). At present, however, a gap still exists between the less and the more intensive approaches to smoking cessation. To fill that void, STIVORO developed a web-based self-help intervention for adult smokers known as the StopSite in 2005. It enables participants to perform quitting activities anonymously, while interacting with others if desired. This conforms to the wishes expressed by most smokers to stop by themselves without professional help [11,12].

A number of web-based self-help programmes for cigarette smoking have become available in the past decade. This is for good reason: the Internet makes it possible to reach vast numbers of smokers, and the programmes could therefore have a major positive impact on public health. Results from several randomised trials have suggested that web-based interventions can promote smoking cessation, especially if the information is appropriately tailored to the users [13-15] and frequent automated contacts with the users are ensured [16]. All in all, the results for web-based self-help programmes are promising, but the number of effectiveness studies is still limited.

The primary objective of this study was to evaluate the effectiveness of the Dutch interactive online self-help intervention (the StopSite) by comparing it to a self-help guide provided on the Internet. A literature review has shown that self-help guides can have small positive effects on smoking cessation in comparison to no intervention [17]. We expected that the StopSite, by virtue of its interactive nature, would result in higher cessation rates than our online guide. Secondary study objectives were to explore possible dose-response effects (whether more visits to the StopSite would result in better outcomes) and whether particular subgroups would benefit more than others. This paper describes the trial protocol and discusses the design, methodology and some methodological issues involved.

## Methods and design

### Study design

The study is a randomised controlled trial. Participants were randomised into two groups: an experimental group performing the web-based interactive self-help intervention and a control group accessing the online self-help guide. The study protocol, interventions and informed consent procedure were approved by the Dutch medical ethics committee METiGG, Chamber North (registration number 5209).

### Inclusion and exclusion criteria

All adults aged 18 and older who were currently smoking cigarettes or rolling tobacco, were willing to quit smoking within three months and had Internet access were eligible for the study. Candidates were excluded if they were already preparing to stop smoking with the support of a coach, a course or pharmacotherapy, or if they were already enrolled in another smoking cessation study.

### Recruitment

Candidates were recruited over a one-year period using advertisements in daily and weekly national or regional newspapers or on the Internet. Enrolment took place via a website that provided information about the study and access to the informed consent form. After returning the signed form by post, candidates received an e-mail telling them how to access and complete an online baseline questionnaire. Those who failed to meet selection criteria were then informed by e-mail.

### Randomisation

The randomisation procedure was automated and was carried out at the individual level after the baseline selection. Randomisation was stratified by gender and age (three age groups: 18 to 29, 30 to 45, and 46 and older). Block randomisation in blocks of two was performed to ensure equal distribution of participants over the two arms of the trial. Participants were informed by e-mail of their allocation to the StopSite or the self-help guide; each received a user name and password to log into the corresponding intervention.

### Interventions

#### The StopSite

The experimental group had access to the StopSite, which has three components. The main component is a self-control programme consisting of exercises based on cognitive-behavioural therapeutic principles. Included are exercises to monitor smoking behaviour, set goals, sustain motivation, analyse risk situations, consider alternative behaviours, and deal with social pressure. The self-control component also includes a device that counts down the days until the chosen quit date, and afterwards displays the number of cigarettes not smoked and the amount of money saved. In the second component, participants can engage in interaction with others in a users' forum or in one-to-one chatrooms with buddies; supportive interaction may motivate them to return to the site regularly. The third component links to a large body of state-of-the-art information about smoking cessation on the STIVORO website .

#### The self-help guide

The comparison group received access to a Dutch online self-help guide developed by STIVORO for smokers who are considering stopping. It is entitled Quit Smoking: Why and How and includes advice on preparing to quit, information about withdrawal symptoms, tips on how to handle difficult moments, and general information about types of support materials. The information is not personalised and not interactive.

#### Support

Both interventions were offered as stand-alone interventions without support from a professional coach. During the trial the participants were allowed to seek additional support if they wished.

### Assessments

Three assessments were made over time: a baseline assessment immediately preceding randomisation and intervention access, and two follow-up assessments at three and six months following a one-month post-baseline grace period. The grace period gave participants time to begin the intervention. At the follow-ups, they were invited by e-mail to complete the online assessment questionnaires, with e-mail reminders sent two weeks later if necessary. To stimulate response, we counted every completed questionnaire as a 'ticket' for a lottery to be held at the end of the study.

### Measures

Outcomes were defined according to the guidelines provided by Mudde et al. [18,19] and Hughes et al. [20] for evaluating smoking cessation interventions.

The primary outcome measure was prolonged abstinence [20] in the past three months. Non-abstinence was defined as having smoked on seven or more consecutive days or two weekends in a row (weekend smokers). Prolonged abstinence was calculated for both follow-up assessments separately and for the two follow-up periods combined. Participants were classified as non-smokers if they were prolonged abstinent at both follow-up assessments.

The secondary outcomes were

1) *point-prevalence abstinence *(no smoking, not even a puff, in the week preceding the questioning)

2) *overall decrease in amounts of tobacco products smoked *(including cigars) between baseline and the follow-up assessments.

### Additional measures

At baseline, we assessed demographics and other potential confounding variables. The potential non-demographic confounding variables as defined by Mudde et al [18] were:

- current stage of change on a readiness-to-change scale. Possible stages for potential quitters are the immotive (no intention to quit), preparation and contemplation stages; the act of quitting is followed by the action stage (less than six months of abstinence) and maintenance stage (six or more months of abstinence)

- attitude towards quitting: expectations regarding effects of smoking cessation in terms of health improvement, sociability, lung cancer risk, relaxation problems, withdrawal symptoms, health of others, and boredom

- social influences on quitting: encouragement by others to quit (5 items) and presence of smokers in daily environment (5 items)

- self-efficacy expectations (abilities to refrain from smoking in difficult circumstances, 6 items).

Other potential confounders were:

- the level of nicotine dependence as measured by the number of cigarettes smoked per day in combination with time to first cigarette (Heaviness of Smoking Index) [21].

- expectations that a web-based self-help programme would be helpful to stop smoking (question constructed by the researchers).

In follow-up assessments, we inquired about the use of professional or other help or support materials (t1 and t2) and participants' evaluation of their assigned intervention (t1). A tracking tool automatically recorded logins to the interventions.

### Sample size

The sample size was based on the expected contrast between the experimental group (StopSite) and the comparison group (self-help guide) in their rates of prolonged abstinence. We expected 5.5% of the comparison group [22] and 11% of the experimental group to report prolonged abstinence at 6-month follow-up. Based on a power of 80% using a two-tailed test with an alpha of .05, at least 353 participants were needed in each condition. Adjusting for an expected 40% dropout at 6 months, we needed a total of 1,104 participants. Sample size was calculated with Stata 7.0/SE.

### Statistical analysis

We used t-tests, chi-square tests and logistic regression (p < .10) to assess whether the randomisation had resulted in two comparable groups at baseline and whether differential loss to follow-up had occurred. We then performed intention-to-treat (ITT) analyses including all randomised participants and classifying those not responding to follow-up questionnaires as smokers (the worst-case scenario); this is a conservative way of handling non-response. We also conducted analyses including only the participants that actually used the assigned intervention. The use of both interventions was monitored by the tracking tool that recorded participant logins; 'use of the intervention' was defined as visiting the StopSite or opening the self-help guide at least once.

Differences between groups at follow-up on dichotomous outcome measures (prolonged abstinence, point-prevalence abstinence and quit attempts) were analysed using chi-square tests or logistic regression, depending on the need to adjust for possible confounders. A linear risk model was used to obtain the risk difference (RD). The number needed to treat (NNT) was calculated as the inverse of the RD (number needed to treat stands for the number of people who must receive the intervention in order for one person to successfully stop smoking).

Between-group differences in tobacco use reduction from baseline to t1 and from baseline to t2 were analysed in linear regression models. Magnitudes of intervention effect were estimated using Cohen's d [23]. Effect sizes were first calculated for each condition separately by subtracting the mean posttest score from the mean pretest score and dividing the result by the standard deviation at pretest. The effect size of the comparison group was then subtracted from that of the experimental group. A difference in d of 0.5 would indicate that the experimental group mean was half a standard deviation greater than the control group mean. Values of d from 0.56 to 1.2 may be regarded as large, from 0.33 to 0.55 as moderate, and from 0 to 0.32 as small [24].

To identify subgroups that particularly benefited from the intervention, we examined the influence that interaction between the smokers' baseline characteristics and the intervention itself might have had on treatment response [25]. In a logistic regression model, the primary outcome (prolonged abstinence) was regressed on dummy-coded baseline characteristics (age and gender), the main intervention effect and the characteristic-by-intervention interaction term. It is the interaction term that is of interest here: it reflects any potential added benefit that the StopSite might have for participants with specific attributes.

To detect possible dose-response relationships, we used a logistic regression model to regress the primary outcome (prolonged abstinence) at t2 on the number of times logged in to the StopSite; only the respondents randomised to the StopSite (the experimental group) were included in the dose-response analyses. Tests were conducted at α = .05 (two-sided) with 95% confidence intervals. Analyses were performed with SPSS version 15.1.

## Discussion

This article describes the protocol of a randomised controlled trial that compared two self-help smoking cessation interventions delivered via the Internet. The primary aim was to assess the effectiveness of StopSite, a Dutch cognitive-behavioural self-help site with interactive elements but without involvement of a coach or therapist. The comparison intervention was a Dutch self-help guide entitled Quit Smoking: Why and How. Both interventions were developed by STIVORO, a Dutch organisation on smoking and health. We expected the StopSite to double the effect of the self-help guide in terms of tobacco abstinence. We did not include a no-treatment control group because of the greater potential for contamination by external interventions; no-treatment controls might be more likely to seek help elsewhere during the study.

Participants were recruited through advertisements in national and regional newspapers and through banners on the Internet. As a consequence, the study is likely to have included disproportionate numbers of smokers with higher motivations to quit; generalisability is therefore restricted to smokers who are already in the preparation or contemplation stage of change. This methodological weakness is also a potential strength, as it probably makes the trial sample a closer reflection of the population being targeted by the intervention: people who are already motivated to stop smoking, but who need some support in doing so and who prefer an intervention without interference of a coach or therapist.

The primary outcome measure was prolonged abstinence from smoking in the six months after the intervention, allowing for a one-month grace period after baseline for performing the intervention. This is a widely accepted, albeit minimum, time frame for smoking cessation trials [26]. The primary outcome, six-month abstinence, was estimated using abstinence measures at two assessment times (three and six months); it was therefore not an ideal measure of prolonged abstinence. We did not perform biochemical verification of self-reported abstinence. That is consistent with recommendations by the Subcommittee on Biochemical Verification of the Society for Research on Nicotine and Tobacco (SRNT), which suggest that biochemical validation may not be necessary or advisable in studies like ours with limited face-to-face contact and with postal, telephone or Internet data collection [27].

The entire trial was conducted via the Internet with the exception of the signed informed consent document that was returned by post. There was no face-to-face or telephone contact between participants and researchers during the trial. The screening and baseline questionnaires, randomisation outcomes, and invitations and reminders for online follow-up assessments were all sent by e-mail. Such an approach ensures a low threshold for study participation, but makes the study susceptible to higher attrition rates due to participant dropout or non-utilisation of the intervention [28].

The blinding of respondents to experimental conditions was not feasible because of the behavioural nature of both interventions. The participants were aware of the intervention to which they were receiving access, but unaware of whether it was the experimental or comparison intervention. No explicit descriptions of the interventions were given before randomisation, thus reducing the chance that participants would feel more disappointed or satisfied with their intervention in relation to the other.

## Competing interests

The authors declare that they have no competing interests.

## Authors' contributions

JK was the principal investigator and wrote the manuscript. MW and AvE obtained funding for the study. HR served as an adviser. BC carried out the recruitment and data collection. SB and all other authors contributed to the manuscript and have approved the final version.

## Pre-publication history

The pre-publication history for this paper can be accessed here:


